# Correlation between the Nonperfusion Area on Ultra-Widefield Fluorescein Angiography and Nonflow Area on Optical Coherence Tomographic Angiography in Retinal Vein Occlusion

**DOI:** 10.1155/2021/5581319

**Published:** 2021-04-30

**Authors:** Jianfeng Huang, Yingyi Lu, Xiaoya Gu, Bodi Zheng, Tong Chen

**Affiliations:** ^1^Medical Doctor, Department of Ophthalmology, Beijing Hospital, National Center of Gerontology, No. 1 Dahua Road Dongcheng District, Beijing, China; ^2^Medical Master, Department of Ophthalmology, Beijing Hospital, National Center of Gerontology, No. 1 Dahua Road Dongcheng District, Beijing, China

## Abstract

**Aims:**

To compare the relationship between the nonperfusion area (NPA) on ultra-widefield fluorescein angiography (UWFFA) and the nonflow area (NFA) on optical coherence tomographic angiography (OCTA) in retinal vein occlusion (RVO).

**Methods:**

Cross-sectional study. 46 eyes of 46 RVO patients who underwent UWFFA and OCTA. NPA and ischemic index (ISI) were quantified on UWWFA. NFA, vessel density (VD) of the superficial capillary plexus (SCP), the deep capillary plexus (DCP), and the size foveal avascular zone (FAZ) on 3 ∗ 3 mm OCTA were measured. The association of the NPA and ISI on UWWFA and the parameters on OCTA were analyzed. Spearman correlation was used for statistical testing.

**Results:**

The NPA and ISI on UWFFA were significantly correlated with the NFA on OCTA in RVO, and *r* values were 0.688 (*p* < 0.01) and 0.680 (*p* < 0.01), respectively. VD in the SCP of the temporal quadrant was negatively correlated with NPA and ISI, and *r* values were −0.346 (*p* < 0.05) and −0.337 (*p* < 0.05), respectively. VD in the DCP of the temporal quadrant was negatively correlated with the NPA, and the *r* value was −0.246 (*p* < 0.05). No significant correlation was found between the NPA and ISI on UWFFA and VD of other quadrants in the SCP or DCP and the FAZ area on OCTA.

**Conclusion:**

NPA in the peripheral retina was correlated with NFA in macula. NFA detected by OCTA could be an indicator of the ischemic status in RVO.

## 1. Introduction

Retinal vein occlusion (RVO) is among the most common type of retinal vascular diseases [1]. RVO can lead to an impairment of blood circulation and retinal ischemia. Extensive peripheral ischemia can lead to retinal neovascularization [2], which can cause vitreous hemorrhage and vision loss. The risk of developing retinal neovascularization is associated with the extent of nonperfusion (NP) [3]. Therefore, adequate measurement of the NP area is critical in the management of RVO. Studies have shown the use of ischemic index (ISI), a ratio of nonperfused retina to total visible retina, as an important assessment in evaluating the ischemic status in retinal vascular diseases including RVO and diabetic retinopathy (DR) [3–5]. Fluorescein angiography (FA) is an established examination to determine the presence of NP. Compared with the 55° FA, the ultra-widefield (UW) FA can display images 200° of the retina, covering more than 80% of the retinal surface in the same angiographic phase [6]. However, FA is an invasive examination and is unrepeatable on the same day or in the short term. Besides, it can cause severe complications such as anaphylaxis, cardiac arrest, and bronchospasm [7]. A noninvasive, safe, and easy-to-manipulate measurement should be developed to evaluate the ischemic status of the retina, or this noninvasive measurement can be used as a predictive factor for a necessary FA.

Optical coherence angiography (OCTA), a noninvasive method to detect the blood flow of retina without intravenous dye injection [8–10], has been widely used in evaluating the microvasculature in the macular area. The nonflow area, vessel density of superficial capillary plexus (SCP), deep capillary plexus (DCP), and area of foveal avascular zone (FAZ) are among the most critical parameters measuerd by OCTA. Studies have indicated that OCTA can enable qualitative and quantitative evaluation of RVO [11–13]. Recently, Glacet-Bernard et al. found that nonperfusion on UWFA correlated with widefield (12 ∗ 12 mm) OCTA [14]. However, there have been no studies on the relationship between the ordinary OCTA (3 ∗ 3 mm or 6 ∗ 6 mm) and UWFA in RVO. Although the field of view of ordinary OCTA is smaller than the widefield one, we considered whether the NP area detected by UWFA is correlated to the nonflow area detected by ordinary 3 ∗ 3 mm OCTA.

In the present study, we sought to compare the correlation between the NP area on UWFA and the nonflow area on OCTA. We aimed to find out whether 3 ∗ 3 mm OCTA can explore the ischemic status of the peripheral retina.

## 2. Methods

### 2.1. Participants

This was a cross-sectional observational study. Patients diagnosed with RVO were recruited in the Department of Ophthalmology at Beijing Hospital, Beijing, from Nov 1, 2019 to Jan 6, 2021. The tenets of the Declaration of Helsinki [15] were followed. Written informed consent to participate in the study was obtained from all individuals after all procedures were explained. Inclusion criteria were the eyes of patients diagnosed with RVO confirmed by fundus examination and FA. Exclusion criteria were the eyes with any history of ocular injury, ocular surgery, retinal laser photocoagulation, and other retinal, optic nerve, and choroidal diseases that may confound the results. Participants with opaque optic media and high refractive error (more than 6 diopters of sphere or more than 3 diopters of cylinder) were also excluded. Only individuals with an OCTA scan quality index above 6 were eligible. OCTA and UWFA were performed on a same day.

### 2.2. UWFA

Standardized UWFFA was performed by an Optos 200Tx (Optos, Dunfermline, UK). Patients' pupils were fully dilated, and UWF pseudocolour images were captured centered on the fovea. Following intravenous administration of fluorescein dye, central UWFFA images were obtained during the early (35 seconds), mid (2 minutes and 30 seconds), and late (5 minutes) phases of the angiogram. After the early phase, the UWFFA images were steered superiorly, inferiorly, temporally, and nasally to allow clear visualization of the boundaries between perfused and ischemic regions.

**Figure 1:**
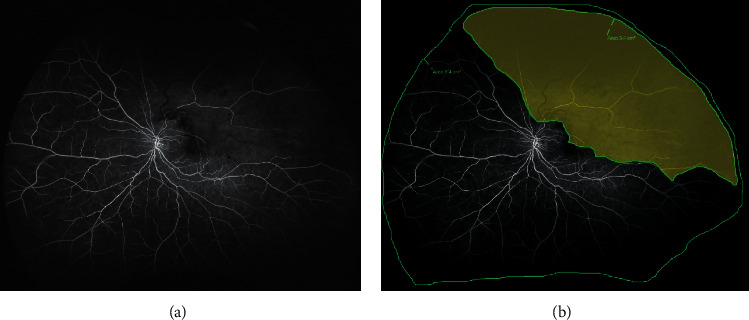
UWFFA images of a left BRVO eye. (a) The original UWFFA image. (b) The yellow mask showed the NPA. The outer circle outlined the total retinal area (8.4 cm^2^) and the inner circle outlined the NPA (3.4 cm^2^).

Images were archived and reviewed using the Optos Advance Software (Optos PLC). The NP area was defined as significant hypofluorecein angiographically. Two trained retinal specialist (LYY and GXY) chose one of the images of the midphase to outline the NP area as well as the total visible retina using the “random pattern mode” in the built-in software (Figure 1). In case of disagreement, a further assessment was performed by an expert grader (C.T). The NP area was calculated automatically by the software. ISI was calculated as the ratio of the NP area to the total visible retinal area.

### 2.3. OCTA

**Figure 2:**
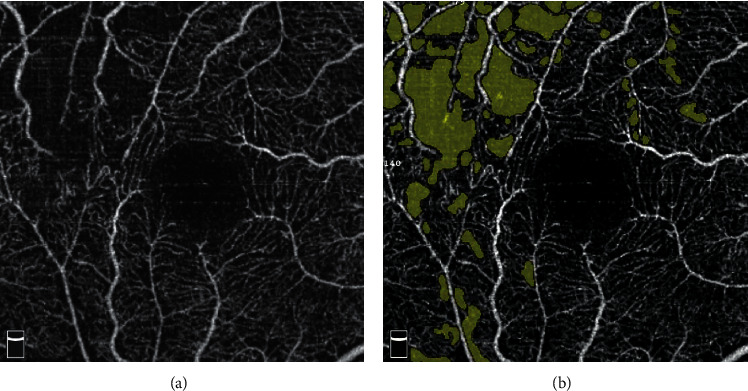
OCTA image of a right BRVO eye. (a) The original OCTA image of the superficial layer. (b) The yellow mask showed the NFA.

**Figure 3:**
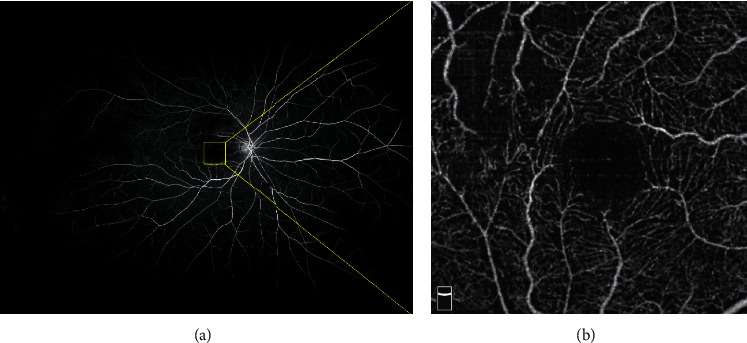
The UWFFA and OCTA images of a same patient with BRVO of the right eye. (a) The UWFFA image. The yellow square on the macula showed the scan area of OCTA. (b) The OCTA image of the same patient.

OCTA images were obtained using the RTVue XR Avanti device with AngioVue 2.0 (Optovue Inc., Fremont, CA, USA). A 3 ∗ 3 mm scan centered on the macular fovea was performed. The built-in Angio Analytics software (version 2017.1.0.155; Optovue, Inc.) was used to evaluate the nonflow area, SCP, DCP, and the FAZ area. SCP and DCP of the whole image, as well as the temporal (T), nasal (N), inferior (I), and superior (S) quadrants in the overlay ETDRS grid and FAZ area were automatically analyzed by the software. The nonflow area was manually labeled by two independent retinal specialists (HJF and ZBD). The labeled nonflow area was counted automatically by the built-in software (Figure 2). The UWFFA and OCTA images of a same patient are shown in Figure 3.

### 2.4. Statistical Analysis

For statistical analysis, IBM SPSS statistics version 21 (IBM SPSS Statistics; IBM Corporation, Chicago, IL, USA) was used. Categorical variables were analyzed using a chi-squared test. The Spearman correlation test was used to examine linear correlations between the OCTA parameters and NP area on UWFFA. Intraclass correlation coefficients (ICCs) were used to assess intergrader reproducibility. The mean of the initial image grading of the two independent graders was used in all subsequent analyses. The independent *t*-test was used to compare continuous variables between groups of the eyes with branch retinal vein occlusion (BRVO) versus those with central retinal vein occlusion (CRVO). A *p* value <0.05 was considered statistically significant.

## 3. Results

UWFFA and OCTA images of 46 eyes from 46 patients were included. 21 eyes were BRVO and 25 eyes were CRVO. The detailed characteristics of the participants are given in Table 1.

### 3.1. Intergrader Correspondence

The ICC value for intergrader agreement on the NP area and ISI on UWFFA and the NF areas on OCTA was 0.950 (*p* < 0.01), 0.953 (*p* < 0.01), and 0.937 (*p* < 0.01), respectively.

### 3.2. NP Area and ISI on UWFFA

Table 2 presents the NP area of all RVO eyes and in BRVO and CRVO eyes. The variables between BRVO and CRVO were compared. Not surprisingly, the NP area of BRVO was significantly less than that of CRVO.

### 3.3. OCTA Parameters of RVO

OCTA parameters including NFA of SCP, VD of SCP and DCP of the whole 3 ∗ 3 mm image, VD of the SCP and DCP of the temporal, superior, nasal, and inferior quadrant of the overlay ETDRS grid, and the FAZ area are given in Table 3. Nonflow area, vessel density of the whole image and of each quadrant, and the FAZ area of CRVO were less than that of BRVO, but only VD of the nasal DCP was significantly different (Table 3).

### 3.4. Correlation between the NP Area and ISI on UWFA and OCTA Parameters of RVO

Positively strong correlation between the NP area and ISI on UWFFA and the NF area on OCTA in RVO was significant. Besides, VD in the SCP of the temporal quadrant was negatively correlated with the NP area and ISI in all RVO eyes and in BRVO eyes. VD in the DCP of the temporal quadrant was negatively correlated with the NP area in all RVO eyes. No significant correlation was found between the NP area and ISI on UWFFA and VD of other zones in the SCP or DCP and the FAZ area on OCTA. Detailed results are given in Table 4.

## 4. Discussion

In this study, we described the correlation between the NP area of the peripheral retina and the nonflow area of macula in RVO. We found that the nonflow area on the 3 ∗ 3 mm OCTA macular image was positively strongly correlated with the NP area and ISI on the UWFA image in RVO, which indicated that the ischemic status of the central retina can be a reflection of the status of the peripheral retina.

RVO is believed to result from compression of the retinal vein by the corresponding retinal arteriole, which is stiffened as a result of underlying hypertension and arteriosclerosis. This, together with damage to the vessel wall, results in thrombus formation [16]. The initial vein occlusion is a precipitating event that causes baseline ischemia and release of the vascular endothelial growth factor (VEGF), which then contributes to progression of NP and thus worsening of ischemia [17]. The worsening of ischemia does not only occur in the periphery but also in the macula, which might be the underlying mechanism for the positive correlation of the NP area on UWFA and the nonflow area on OCTA found in our study. Sawada et al. showed that widefield OCTA can detect NP with high sensitivity and specificity when compared with UWFA in diabetic retinopathy (DR) [7]. Marco Pellegrini et al. found that widefield OCTA can detect more NPA than 55° FA in retinal vascular diseases [18]. Takao Hirano et al. showed in their study that there was no significant difference in extent of NPA or number of NVs between FA and widefield OCTA [19]. Other previous studies also demonstrated that conventional 3 ∗ 3 or 6 ∗ 6 mm OCTA can detect NP to nearly the same extent as FA and in DR [10, 20, 21]. Our study had the similar result that the nonflow area on conventional 3 ∗ 3 mm OCTA image was positively correlated to the NP area of peripheral retina. Compared to the automatically calculated parameters such as vessel densities of the SCP/DCP and FAZ area, the manually labeled nonflow area in macula was not taken into account in most of the previous studies, but the nonflow area measured by OCTA was also an important parameter for the assessment and of microvasculature in the macular area, as Kuonen A et al. demonstrated in their study in which the nonflow area was found to be related to DR severity [22].

We found that vessel densities of the SCP, DCP, and the FAZ area were not significantly correlated with the NP area in RVO except for the temporal vessel density of the SCP and DCP. Previous studies reported decreased vessel densities of SCP and/or DCP in the eyes with either BRVO or CRVO compared with normal eyes [23–25]. Recently, Glacet-Bernard et al. found in their study that the ISI on UWFA and vessel densities in the superficial and deep plexus on widefield OCTA (12 ∗ 12 mm) correlated significantly [14]. In contrast to their finding, our study indicated that the reduction of vessel density in the whole macula area detected by OCTA was not correlated with the NP area in peripheral retina in RVO. The possible reason might lie on the different severities of RVO patients recruited as well as different OCTA devices and scanning field (3 ∗ 3 mm vs. 12 ∗ 12 mm), but the underlying mechanism should be discussed by further studies. Interestingly, we found the temporal VD of the SCP and DCP were correlated with the NP area and ISI on UWFFA in all RVO eyes and in BRVO eyes. The possible explanation for this might be all of the BRVO eyes recruited in our study were temporal BRVO; therefore, the temporal microvasculature of the macula could be more affected than the other quadrants.

Both the NP area on UWFFA and the nonflow area on OCTA were calculated by the built-in software in the devices in our study. Although the NP area on UWFFA and the NF area on OCTA were labeled manually, the intergrader agreement was excellent. UWFFA can provide larger angle of the retina than the conventional one, but the distortion from 3D retina to 2D image is still a problem. Many of the previous studies have developed several ways to reduce the distortion on calculating the precise NP area on UWFFA [3, 6, 26], but currently, there is no gold standard for measuring the NP area and ISI by UWFFA. We used the same built-in software as Glacet-Bernard A et al. used because the built-in software now can automatically correct the nonlinear warping at the periphery of the image [14]. The images analyzed in the present study were central rather than montaged as research studies before showed that the NP area and ISI for central and montaged images were not significantly different for any retinal zone [6, 27].

There were several limitations of our study. First, the number of cases was limited. Second, the NP area on UWFFA and nonflow area on OCTA were manually labeled, even though the intergrader agreement was excellent and error caused labelling still existed. Third, most of the patients recruited in our study were clinically severe RVO that the NP area was larger than some of the previous publications. RVO with milder condition and smaller NP area should be recruited in the future study. Fourth, the OCTA scan was 3 ∗ 3 mm in our study, which provided less information than the widefield OCTA scans. Despite these limitations, the results of our study provided a new insight on the relationship between the NP area in the peripheral retina and the NF area in macula.

## 5. Conclusion

In conclusion, the finding from our results suggested that the nonperfusion area in the peripheral retina was correlated with the nonflow area in macula. Nonflow area detected by OCTA could be an indicator of the ischemic status in RVO.

## Figures and Tables

**Table 1:** Demographic characteristics of patients with RVO.

Characteristics	Total	BRVO	CRVO	*p* value
Patients (no.)	46	20	26	
Eyes (no.)	46	20	26	
Age (y)	61.3 ± 11.7	64.2 ± 8.8	59.1 ± 13.1	0.137
Sex				0.234
Female, no. (%)	23 (50%)	12 (60%)	11 (42.3%)	
Male, no. (%)	23 (50%)	8 (40%)	15 (57.7%)	

**Table 2:** NP area and ISI on UWFA of RVO.

	Total	BRVO	CRVO	*p* value (BRVO vs. CRVO)
NP area (cm^2^)	2.58 ± 1.72	1.88 ± 1.15	3.12 ± 1.91	0.014
ISI	0.31 ± 0.21	0.22 ± 0.14	0.37 ± 0.23	0.017

**Table 3:** OCTA parameters of RVO.

	Total	BRVO	CRVO	*p* value
Nonflow areas (mm^2^)	2.74 ± 1.71	2.27 ± 1.41	3.10 ± 1.86	0.10
SCP (whole image)	40.37 ± 5.40	41.65 ± 4.10	39.39 ± 6.13	0.16
SCP (temporal)	39.51 ± 7.59	40.48 ± 7.33	38.76 ± 7.85	0.45
SCP (superior)	42.56 ± 7.72	43.93 ± 7.96	41.52 ± 7.53	0.31
SCP (nasal)	41.77 ± 6.70	42.92 ± 5.48	40.91 ± 7.49	0.34
SCP (inferior)	42.39 ± 8.37	45.00 ± 6.88	40.37 ± 8.97	0.06
DCP (whole image)	40.67 ± 6.25	41.84 ± 5.50	39.77 ± 6.74	0.27
DCP (temporal)	42.43 ± 8.38	44.13 ± 8.05	41.14 ± 8.54	0.23
DCP (superior)	43.35 ± 7.56	45.89 ± 7.80	41.42 ± 6.92	0.05
DCP (nasal)	44.12 ± 6.58	46.59 ± 6.29	42.28 ± 6.29	0.03
DCP (inferior)	42.86 ± 8.62	44.22 ± 8.48	41.82 ± 8.76	0.36
FAZ area (mm^2^)	0.37 ± 0.25	0.44 ± 0.30	0.32 ± 0.19	0.14

**Table 4:** Correlation between the NP area and ISI on UWFFA and OCTA parameters of RVO.

	Total		BRVO		CRVO	
	NPA	ISI	NPA	ISI	NPA	ISI
Nonflow areas (mm^2^)	0.688^*∗∗*^	0.680^*∗∗*^	0.687^*∗∗*^	0.674^*∗∗*^	0.656^*∗∗*^	0.653^*∗∗*^
SCP (whole image)	−0.205	−0.184	−0.062	−0.024	−0.234	−0.210
SCP (temporal)	−0.346^*∗*^	−0.337^*∗*^	−0.522^*∗*^	−0.502^*∗*^	−0.203	−0.183
SCP (superior)	−0.176	−0.146	−0.040	−0.011	−0.148	−0.119
SCP (nasal)	−0.161	−0.148	−0.092	−0.071	−0.191	−0.158
SCP (inferior)	−0.194	−0.181	0.083	0.141	−0.201	−0.190
DCP (whole image)	−0.246^*∗*^	−0.219	−0.260	−0.212	−0.123	−0.101
DCP (temporal)	−0.294	−0.285	−0.224	−0.227	−0.164	−0.155
DCP (superior)	−0.087	−0.050	0.002	0.025	0.128	0.159
DCP (nasal)	−0.222	−0.198	−0.124	−0.109	0.002	0.045
DCP (inferior)	−0.109	−0.084	0.062	0.113	−0.061	−0.032
FAZ area (mm^2^)	−0.197	−0.171	−0.047	−0.030	−0.178	−0.153

^*∗∗*^ < 0.01; ^*∗*^ < 05.

## Data Availability

The data used to support the findings of this study are available from the corresponding author upon request.
